# Looking after YOU

**Published:** 2015

**Authors:** Heather Machin

**Affiliations:** Registered Nurse and Consultant: Fred Hollows Foundation NZ, Aukland, New Zealand. hmachin@hollows.org.nz

**Figure:**
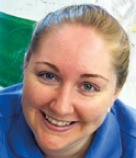
Heather Machin

It sounds silly, but we often spend so much time caring for our patients and each other that we forget to care for ourselves. If we don't look after ourselves it could put us at risk and could lead to a short-term or permanent inability to do our job. We need to do whatever we can as health care professionals to demonstrate to our patients, through our own example, what it means to live a healthy and enjoyable life – there is no better health promotion tool than that!

Here isa list of some of the things you can do to take good care of YOU.

**Have your eyes checked.** It is important to have them checked regularly. Your eyesight is important because you need to perform several specialised tasks – be it reading labels on a drug bottle or seeing a suture clearly enough to be able to load it onto a needle-holder. You work in eye care so you have no excuse – get your eyes checked.

**Sleep.** Make sure you get a good night's sleep every night. This includes about 6–8 hours of solid sleep. This is particularly important if you also do night duty because your sleep patterns may not be routine.

A good night's sleep will help to ensure that you have energy to perform your daily tasks. It also increases your brain's ability to focus on difficult and complex tasks and issues.

**‘Make sure you get a good night's sleep every night.’**

**Body care.** Make sure you comply with your hospital's policy on lifting and moving objects correctly. If you hurt a part of your body while at work you are at risk of having a permanent injury, which could be painful and reduce your job options. There is lots of online information about this subject. You can search on the internet for ‘ergonomics’, which is the study of the body's movement or try ‘workplace or occupation health and safety’ or ‘manual handling’.

**Practise mindfulness.** Try to have some alone time, by yourself, from time to time, so you can recharge your energy levels and take back your enjoyment and love for life. Search on the internet for ‘mindfulness’ – there are many websites which offer practical guidance to this approach, which has been shown to improve mental wellbeing.

**Hygiene.** Make sure you wash regularly, as you work in a hospital and it is important that you are not carrying harmful organisms to and from your workplace and home.

**Figure:**
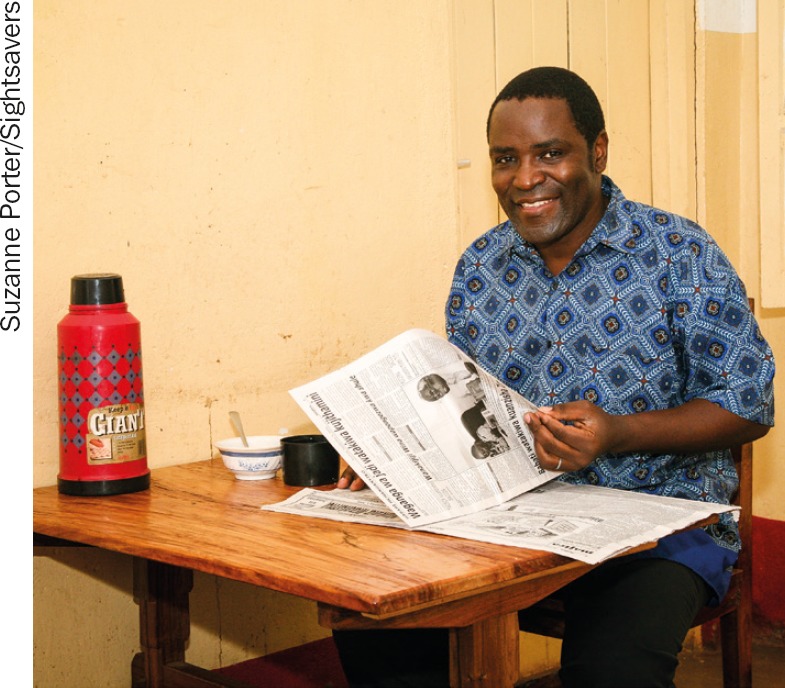
It is important to have regular breaks during a busy day in the clinic. TANZANIA

**Keep fit.** The human body is designed to move, so make sure you are moving your body regularly. This could include a walk to and from work.

**Spend time with your family and friends.** Work is very important and helps bring money into the home. While it remains important, don't forget to spend time with your family and friends whenever you can. Jobs will come and go but family and friends remain. They need you as much as you need them. Prioritise them!

**Eat well.** Health care workers are notorious for grabbing quick snacks, missing lunch and going without a drink for hours. This is not good. Drink plenty of water and try and have lunch or some snacks every day.

**Stay safe.** If you are travelling to and from work after dark you need to take care of your own safety and make sure you are not in danger. Hospitals are easy targets for crime so never place yourself in danger.

**Travel responsibly.** If you travel a lot for work, be mindful of safety, regular food, exercise and rest.

**Look after your professional registration.** Find out what your professional association (i.e. medical or nursing council) requires of you to maintain your professional license and make sure you comply with those requirements.

**Join an association.** Networking with others in your profession is very helpful. It allows you to share your stories and learn from others’ experiences. It is a good way to stay aware of changes and new opportunities in your area of work.

